# Complex pattern of interaction between *in utero* hypoxia-ischemia and intra-amniotic inflammation disrupts brain development and motor function

**DOI:** 10.1186/1742-2094-11-131

**Published:** 2014-08-01

**Authors:** Lauren L Jantzie, Christopher J Corbett, Jacqueline Berglass, Daniel J Firl, Julian Flores, Rebekah Mannix, Shenandoah Robinson

**Affiliations:** 1 Departments of Neurology and Neurosurgery, F.M. Kirby Center for Neurobiology, Boston Children’s Hospital, Harvard Medical School, 300 Longwood Avenue, Boston, MA 02115, USA; 2 Current address: Department of Pediatrics, UNM, Office of Pediatric Research, MSC10 5590, 1 University of New Mexico, Albuquerque, NM 87131, USA

**Keywords:** Preterm, Hypoxia-ischemia, Inflammation, Lipopolysaccharide, Erythropoietin, Prematurity, Myelin, Neurofilament, Gait, Motor deficit

## Abstract

**Background:**

Infants born preterm commonly suffer from a combination of hypoxia-ischemia (HI) and infectious perinatal inflammatory insults that lead to cerebral palsy, cognitive delay, behavioral issues and epilepsy. Using a novel rat model of combined late gestation HI and lipopolysaccharide (LPS)-induced inflammation, we tested our hypothesis that inflammation from HI and LPS differentially affects gliosis, white matter development and motor impairment during the first postnatal month.

**Methods:**

Pregnant rats underwent laparotomy on embryonic day 18 and transient systemic HI (TSHI) and/or intra-amniotic LPS injection. Shams received laparotomy and anesthesia only. Pups were born at term. Immunohistochemistry with stereological estimates was performed to assess regional glial loads, and western blots were performed for protein expression. Erythropoietin ligand and receptor levels were quantified using quantitative PCR. Digigait analysis detected gait deficits. Statistical analysis was performed with one-way analysis of variance and *post-hoc* Bonferonni correction.

**Results:**

Microglial and astroglial immunolabeling are elevated in TSHI + LPS fimbria at postnatal day 2 compared to sham (both *P* < 0.03). At postnatal day 15, myelin basic protein expression is reduced by 31% in TSHI + LPS pups compared to shams (*P* < 0.05). By postnatal day 28, white matter injury shifts from the acute injury pattern to a chronic injury pattern in TSHI pups only. Both myelin basic protein expression (*P* < 0.01) and the phosphoneurofilament/neurofilament ratio, a marker of axonal dysfunction, are reduced in postnatal day 28 TSHI pups (*P* < 0.001). Erythropoietin ligand to receptor ratios differ between brains exposed to TSHI and LPS. Gait analyses reveal that all groups (TSHI, LPS and TSHI + LPS) are ataxic with deficits in stride, paw placement, gait consistency and coordination (all *P* < 0.001).

**Conclusions:**

Prenatal TSHI and TSHI + LPS lead to different patterns of injury with respect to myelination, axon integrity and gait deficits. Dual injury leads to acute alterations in glial response and cellular inflammation, while TSHI alone causes more prominent chronic white matter and axonal injury. Both injuries cause significant gait deficits. Further study will contribute to stratification of injury mechanisms in preterm infants, and guide the use of promising therapeutic interventions.

## Background

In 2010, there were approximately 15 million preterm births (gestation <37 weeks) worldwide. In most middle- and high-income countries, preterm birth is the leading cause of childhood death [1,2]. Beyond mortality, preterm birth can have extensive impact on neurodevelopment, with increased risks of cerebral palsy, impaired learning, vision and hearing loss, epilepsy, psychiatric disorders and poor physical health. Together, these deficits contribute to the prematurity-related burden of chronic disease in adulthood [2,3]. Infants born extremely preterm (<28 weeks gestational age) are particularly prone to chronic neurological deficits, with up to one half experiencing significant cognitive delay and behavioral problems [4,5]. To identify and refine targets for new therapeutic interventions, a clearer understanding of how perinatal insults translate to impaired neurodevelopment is imperative.

Central nervous system (CNS) injury associated with preterm birth has multiple causes [6,7]. The risks of neurological deficits are higher in preterm infants who are also small for gestational age (weight <10% expected for age) or who have associated perinatal infection [4,8-11]. Maternal smoking and higher placental resistance indices are associated with increased risk of preterm or small for gestational age births [12,13], and signs of maternal vascular underperfusion in the placenta of extremely low gestational age newborns (ELGANs) are associated with cerebral palsy in childhood [14]. Conversely, acute chorioamnionitis is the most common abnormality found in placentas from extremely preterm births [15]. Detailed analyses of placentas from ELGANs show inflammation is more commonly associated with spontaneous early birth, whereas less inflammation was present in placenta from births induced for maternal health such as pre-eclampsia [16]. Intrauterine infection increases the risk of cerebral palsy in children born preterm [10,17,18], and recent well controlled studies have confirmed that chorioamnionitis is associated with cerebral palsy in children who were born very preterm [19], and with cognitive impairment at 2 years in ELGANs [20]. Together, these human studies of extremely preterm birth reveal a strong correlation between *in utero* bacterial inflammation and hypoxia-ischemia (HI) and subsequent impaired brain development. These studies also suggest that various types of injury, alone or in combination, may impact the developing CNS via different mechanisms, and thus require tailored neonatal cocktails of emerging neuro-reparative interventions.

Systemic perinatal HI and inflammation appear to act in concert to potentiate CNS damage from preterm birth. For example, early hemodynamic disturbances were present in very preterm newborns exposed to chorioamnionitis and elevated cord blood cytokines [21], or premature rupture of membranes [22], but not in their peers without these signs. In another prospective study, the risk for abnormal neurologic outcome at 2 years was highest for very preterm infants exposed to both histologic chorioamnionitis and additional placental perfusion defects [23]. Preclinical studies have demonstrated the cumulative nature of HI and inflammatory insults to the developing brain [24-31], but have not mimicked the combined prenatal global HI injury with intrauterine bacterial inflammation that directly models the extreme preterm human condition. Significant overlap appears to be present in the activation of aberrant signaling pathways induced by HI and bacterial inflammation. Our limited understanding of the mechanisms underlying *in utero* insults that adversely affect neurodevelopment following extremely preterm birth hinders the rational design and use of neonatal interventions to mitigate early CNS injury. In particular, the impact on neurodevelopment of coincident prenatal bacterial inflammation with global HI remains to be defined for ELGANs.

Both the fetal CNS and immune system are developmentally regulated. To more accurately mimic the brain injury from extremely preterm birth in humans, we developed a clinically relevant rat model of *in utero* brain injury that includes HI from placental perfusion dysfunction plus inflammation from bacterial chorioamnionitis to investigate the response of the developing brain to HI alone, inflammation alone or a dual HI-inflammatory injury. Due to its high molecular weight, lipopolysaccharide (LPS), the cell wall component of Gram negative bacteria that initiates the inflammatory cascade, does not cross the healthy placenta well, and the maternal LPS-induced inflammatory response likely differs from the immature fetal response. Because LPS has also recently been found in cord blood in preterm infants with chorioamnionitis [32], and microbial invasion of the placenta is present in extremely preterm births [10], here intra-amniotic injection of LPS was used to model the fetal inflammatory response to bacteria. Transient systemic HI (TSHI) via uterine artery occlusion on embryonic day 18 (E18), neurodevelopmentally equivalent to approximately 25 weeks estimated gestational age (EGA) in humans, induces white matter loss, gliosis and functional deficits in adult rats [33], and premature loss of the subplate [34] that mimics many aspects of the pathological abnormalities observed in human post-mortem samples from preterm infants with white matter lesions [35,36]. Prenatal TSHI in rats also induces marked upregulation of the erythropoietin (EPO) receptor in the neonatal brain, leading to a mismatch of EPO ligand:receptor signaling on neurons and oligodendrocytes [37,38]. We hypothesized that prenatal TSHI plus intra-amniotic LPS administration (TSHI + LPS) in rats would induce different cellular, biochemical and functional effects on the offspring, compared to prenatal LPS alone or TSHI alone. Specifically, we found that the combination of TSHI + LPS leads to neonatal encephalomalacia and ventriculomegaly, acute chronic microgliosis and astrocytosis in white matter, and decreased myelin basic protein (MBP) expression, neurofilament (NF) ratio and motor deficits in juvenile postnatal day 28 (P28) rats. The various prenatal injuries also differentially affect the developmental regulation of EPO ligand and receptor transcription levels. This clinically relevant model will illuminate dissection of the molecular pathways critical to the development of safe, effective interventions for these vulnerable neonates.

## Methods

### Prenatal injury model

**Figure 1:**
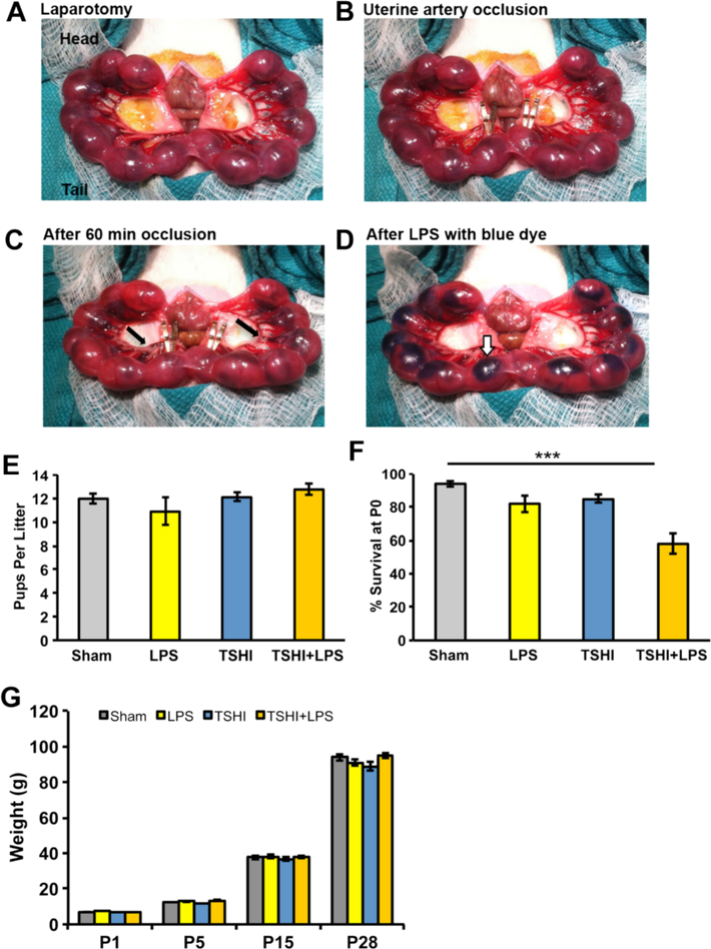
**Combined intrauterine transient hypoxia-ischemia and intra-amniotic lipopolysaccharide in embryonic day 18 rats. (A)** Under isoflurane anesthesia, uterine arteries are exposed via midline laparotomy. **(B)** Occlusion and transient systemic hypoxia-ischemia (TSHI) is initiated with placement of aneurysm clips on uterine arteries. **(C)** After 60 minutes of occlusion, blood is darker in both the vessels and the fetuses (black arrows) and clips are removed. **(D)** Lipopolysaccharide (LPS), with Evans blue dye added to improve visualization (white arrows), is injected into each amniotic sac. Shams undergo laparotomy and 60 minutes of anesthesia. **(E)** No significant difference in litter size was observed among the various insult types (n = 8 to 37, mean ± SEM). **(F)** Fetal loss at postnatal day 0 (P0) is significantly increased in TSHI + LPS insult groups compared to shams (n = 8 to 37, ***P < 0.001). **(G)** The various prenatal insults did not cause significant differences in weight at P28 (n = 8 to 30).

All procedures were performed in accordance with the NIH Guide for the Care and Use of Laboratory Animals and with the approval of the Institutional Animal Care and Use Committee at Boston Children’s Hospital. Under isoflurane anesthesia, a laparotomy was performed on pregnant Sprague–Dawley rats on E18 (Figure 1A). For TSHI, uterine arteries were occluded (Figure 1B) and after 60 minutes the clips were removed (Figure 1C). For the combined injury, following 60 minutes of TSHI, 4 μg sterile LPS (LPS 0111:B4, Sigma, St. Louis, MO) mixed with diluted Evans blue dye (Sigma) was injected into each amniotic sac (Figure 1D). For LPS alone, LPS was injected without transient uterine artery occlusion. For sham controls, the laparotomy was performed and uterine horns were exposed for 60 minutes, without artery occlusion or LPS injection. Thus, all dams experienced an equivalent time of laparotomy under anesthesia. Pups were born at term (E22) and matured with their respective dams. Litter size was recorded. Pups were weaned at P21. Pups were weighed at the ages noted, and gender was recorded. Overall, 12 sham, 16 TSHI, 7 LPS and 18 TSHI + LPS dams were used.

### Immunohistochemistry

Following a lethal dose of sodium pentobarbital, pups were perfused at P2 (n = 4 to 6 per group) and P15 (n = 3 to 6 per group) and brains were processed for immunohistochemistry. Serial 20 μm coronal brain sections were cut on a cryostat (Leica, Buffalo Grove, IL) from the anterior frontal lobes through the posterior extent of the dorsal hippocampus with sections mounted on slides using stereology protocols. Blocking solution contained 10% normal goat serum and 0.1% triton in phosphate-buffered saline. For microglia (ionized calcium-binding adapter molecule 1; Iba1) and astrocyte (glial fibrillary acidic protein; GFAP) immunohistochemistry, sections were incubated with hydrogen peroxide treatment, block, and then primary antibodies (Iba1, 1:500, Wako, Cambridge, MA; and GFAP, 1:500, Dako, Carpenteria, CA) overnight on sections at 4°C. The following day sections were washed, and incubated sequentially with appropriate biotinylated secondary antibodies (1:200, Vector, Southfield, MI), Vectastain (Vector), and diaminobenzadine, and mounted with Permount. Adjacent sections were stained with H&E and mounted with Permount. For MBP immunolabeling, after blocking, sections were incubated with anti-MBP antibodies (1:1000, Covance, Dedham, MA) overnight. The following day, sections were washed and incubated with Alexa-Fluor 568 conjugated anti-mouse secondary antibodies (Life Technologies, Grand Island, NY) for 1 hour. Sections were then washed and coverslipped in an aqueous, DAPI-containing mounting medium. Images were photographed using consistent settings on a Leica microscope and compiled in Photoshop (Adobe, San Jose, CA).

### Stereological estimates

All sections were coded prior to analysis to blind the observer performing the counting. A Leica microscope with a motorized stage and electronic microcator was used with Stereologer software (Stereology Resource Center, Tampa, FL) to perform the analyses. Lateral ventricular volume was measured on P2 and P15 coronal sections visualized with H&E using Cavalieri’s method with the Stereologer software. Object area fraction and volume probes were used to calculate load and to quantify the amount of Iba1 + microglia and GFAP + astrocytes as these cells change in number, size and morphology following injury and through normal development. Estimates of the load of each antigen were obtained from 20 μm coronal sections using a thin section modification of the optical fractionator method [39,40]. Regions of interest (ROI) at P2 and P15 were defined based on anatomical landmarks on coronal sections; the corresponding measurements from the adult brain in Paxinos are noted only for the reader’s orientation [41]. In this study for the fimbria, the borders were the following: anterior (anterior margin of the hippocampus, bregma -1.80), posterior (anterior margin of the fasciola cinereum in the mesial hippocampus, bregma -2.92), medial (thalamus), lateral (where the fimbria abuts the lateral ventricle), superior (hippocampus), and inferior (stria terminalis). The volume of the ROI was calculated using Cavalieri’s method. Briefly, the software superimposed a lattice of regularly spaced plus-signs over the ROI, and the ROI was outlined on every eighth section for P2 and every sixth section for P15. The number of cells within each systematically spaced unbiased sampling frame was counted, and the coefficient of error were calculated and all <10%. At the completion of the stereological analyses, the samples were decoded, and mean and SEM of load were calculated.

### Quantitative PCR

Rat pups (n = 4 to 8/group/age) were euthanized by decapitation and P2 forebrain and P15 white matter was dissected for transcriptional analyses using quantitative PCR. Gene-of-interest primers and cDNA synthesized from 0.9 μg RNA were added to Sybr green universal MasterMix (Kapa, Wilmington, MA), and run in triplicate on a LifeTech Step-One Plus (Life Technologies, Grand Island, NY). To standardize transcripts from samples between experiments, cycle thresholds from samples of interest were compared to cycle threshold values from pooled E19 frontal cortex samples from naïve pups, with gene-of-interest transcription normalized to an 18 s endogenous control. The primers used (all from IDT Technologies, Coralville, Iowa) are listed in Table 1.

**Table 1:** List of primers used for quantitative PCR

EPOR Forward:	GAC CCC AGC TCT AAG CTC CT
EPOR Reverse:	AGC CCC CTG AGC TGT AAT CT
EPO Forward:	GCT CCA ATC TTT GTG GCA TC
EPO Reverse:	ATC CAT GTC TTG CCC CCT A
18s Forward:	TCC CTA GTG ATC CCC GAG AAG T
18s Reverse:	CCC TTA ATG GCA GTG ATA GCG A

### Western blots

Western blots were performed on tissue homogenate from microdissected white matter collected at P15 and P28 following decapitation. After sonication, whole cell proteins were isolated and a Bradford protein assay was performed. Protein (30 μg) was loaded on 4 to 20% precast Tris HCl or 4 to 12% Bis Tris gels (BioRad, Hercules, CA). Following transfer to polyvinylidene fluoride (PVDF) membranes, membranes were blocked and incubated overnight at 4°C in anti-MBP (1:500, Millipore, Darmstadt, Germany), anti-NF (1:1000, SMI-312, Covance), anti-phosphoNF (1:500, Millipore) or anti-actin (1:5000, Sigma) antibodies. Following washes and incubation with species-appropriate HRP-conjugated secondary antibodies, membranes were washed and incubated in femto-West ECL and developed on a GE-LAS 4000 Digital image reader (GE Healthsciences, Pittsburgh, PA). Resultant bands were quantified using GE ImageQuant software (GE Healthsciences) and standardized to actin to confirm equal protein loading among lanes.

### Functional assessment

On P28 the gait of sham control (n = 18), LPS (n = 13), TSHI (n = 14) and TSHI + LPS (n = 21) rats of both sexes were quantified using the DigiGait™ Imaging System (Mouse Specifics, Inc., Boston, MA) [34]. All DigiGait testing was performed in a blinded fashion and all settings, including camera, lighting, shutter and belt speed were optimized before experimental testing. Briefly, a video camera mounted below a motorized transparent treadmill belt captured the ventral side of each rat while walking. Rats were placed on the treadmill which was rapidly accelerated to 30 cm/second, the speed at which the data is captured for analysis. The recorded output, including digital paw prints and dynamic gait signals yielding the temporal record of paw placement relative to the treadmill belt, was analyzed using the DigiGait analysis software v.12.2 (Mouse Specifics, Inc.). Numerous postural and kinematic metrics of gait dynamics were determined by dissecting the time each limb spent in various portions of the walking phase, including paw area, paw placement during stance, stride length, stepping frequency, and stride pattern [42]. Time was measured in each phase of the walking stride (brake, propel and swing) and gait consistency and coordination were assessed. Forelimb and hindlimb data were taken as the average measurement taken across the respective left and right limbs [34,43].

### Statistics and quantification

Data are presented as mean with SEM. Statistical analyses comparing treatment groups were performed using a one-way analysis of variance (ANOVA) with Bonferroni’s correction for multiple pairwise comparisons using IBM Statistics 21 (Armonk, NY, formerly SPSS), with *P* < 0.05 considered significant.

## Results

### Impact of prenatal injury on overall health

We first evaluated fetal survival and pup growth following the different types of insults. No differences in litter size were observed at the time of laparotomy on E18 between the different lesion types (Figure 1E). Following prenatal sham, LPS, TSHI, and TSHI + LPS insults on E18, fetal loss at birth (P0) was markedly increased in TSHI + LPS to 42% (one-way ANOVA, *P* < 0.001), compared to 18% for LPS alone, 15% for TSHI alone, and 6% after sham (Figure 1F). All pups that survived to P1 remained alive. Pups were weighed on P1, P5, P15 and P28, and no significant weight differences were observed between lesion types throughout the investigated time course (Figure 1G). Thus, potential differences in functional performance were not related to overall growth.

### Prenatal injury impacts neonatal ventricular size

**Figure 2:**
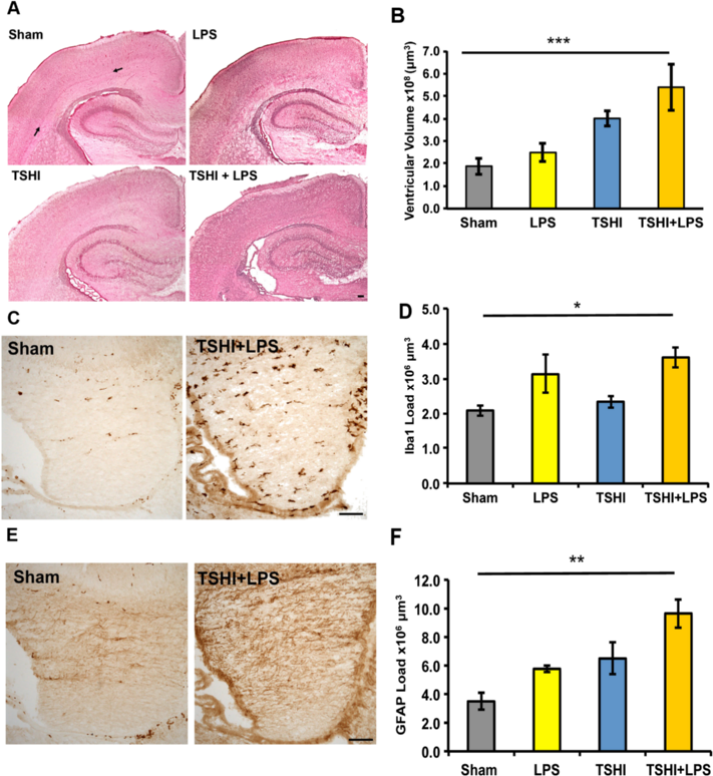
**Prenatal transient systemic hypoxia-ischemia and/or intra-amniotic lipopolysaccharide leads to histological abnormalities on postnatal day 2. (A)** Coronal sections from shams and each of the three insults (lipopolysaccharide (LPS) alone, transient systemic hypoxia-ischemia **(**TSHI) alone and TSHI + LPS) stained with H&E at P2 show the subplate (arrows) is present in shams, but less visible after the insults. Ventriculomegaly and encephalomalacia are more prominent following prenatal TSHI and TSHI + LPS, compared to shams and LPS alone. Scale bar = 100 μm. **(B)** Lateral ventricular volume is significantly larger in TSHI + LPS pups compared to shams (n = 3-5, ***P < 0.001). **(C)** Representative photomicrographs depict increased ionized calcium-binding adapter molecule 1 (Iba1) immunoreactivity in the fimbria of TSHI + LPS pups. Scale bar = 100 μm. **(D)** Similarly, Iba1 + load is significantly increased in TSHI + LPS pups compared to sham (n = 3-4, *P < 0.05). **(E)** In the fimbria, representative photomicrographs show glial fibrillary acidic protein (GFAP) immunoreactivity is also increased in TSHI + LPS pups. Scale bar = 100 μm. **(F)** GFAP + load in the fimbria is significantly increased in TSHI + LPS pups compared to sham (n = 3-4, **P < 0.01).

Neonatal ventriculomegaly is a common finding in neonates born preterm with CNS injury. Histological examination of H&E stained brains at P2 revealed significant ventriculomegaly and encephalomalacia following TSHI and TSHI + LPS (Figure 2A). Specifically, at P2 both TSHI and TSHI + LPS brains had significantly larger lateral ventricles (4.0 ± 0.4 × 10^8^ μm^3^, n = 5, *P* = 0.001 and 5.0 ± 0.1 × 10^8^ μm^3^, n = 3, *P* = 0.01, respectively) compared to shams (1.9 ± 0.4 × 10^8^ μm^3^, n = 3, Figure 2B). None of the rats at any age developed hydrocephalus with macrocephaly. Note also that the subplate is visible in the sham at P2 but is less apparent after the TSHI and TSHI + LPS lesions (arrows in Figure 2A). With or without the addition of LPS, prenatal TSHI caused macroscopic cerebral changes at P2 consistent with those observed in ELGANs with CNS injury.

### Early glial response is altered by prenatal injury

Gliosis is another hallmark of CNS injury from preterm birth. Immunolabeling and stereological analyses of P2 brains revealed significantly increased Iba1 + and GFAP + loads in the fimbria of TSHI + LPS animals compared to sham white matter, indicative of an acute cellular inflammatory response with microgliosis and astrocytosis, respectively (Figure 2C,E). Specifically, Iba1 + load in TSHI + LPS animals was 3.5 ± 0.3 × 10^6^ (n = 3) compared to 2.1 ± 0.2 × 10^6^ in sham animals (n = 3, *P* = 0.025, Figure 2D). Similarly, GFAP + load in TSHI + LPS animals was also significantly increased (9.7 ± 1.0 × 10^6^ versus 3.5 ± 0.6 × 10^6^, n = 3, *P* = 0.003, Figure 2F). The combined prenatal TSHI + LPS on E18 induced a glial response that persisted 5 to 6 days until P2, equivalent to approximately 32 weeks EGA in a human infant.

### Myelin basic protein expression is reduced at postnatal day 15 after prenatal injury

**Figure 3:**
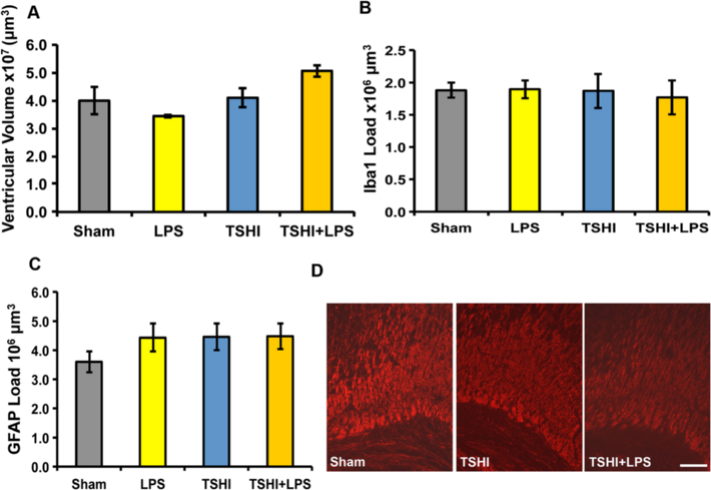
**Myelin basic protein expression is decreased on postnatal day 15 following prenatal transient systemic hypoxia-ischemia and/or lipopolysaccharide injury. (A)** At postnatal day 15 no difference in lateral ventricle volume is observed (n = 3-6). **(B,C)** Similarly, stereological investigations reveal ionized calcium-binding adapter molecule 1 (Iba1) + and glial fibrillary acidic protein (GFAP) + load in the fimbria are not significantly different following the different types of prenatal insults (n = 3-4). **(D)** Myelin basic protein immunolabeling shows reduced protein expression in the periventricular white matter of transient systemic hypoxia-ischemia (TSHI) and TSHI + lipopolysaccharide (LPS) pups compared to shams. Scale bar = 100 μm.

To determine if the ventriculomegaly and gliosis persisted beyond the acute period of injury, stereological analyses of immunolabeling of Iba1 and GFAP were performed at P15, approximately 19 days following the E18 lesion. While analyses revealed no significant differences in ventricular volume (Figure 3A), Iba1 + load (Figure 3B) or GFAP + load (Figure 3C) in the fimbria, a significant reduction in MBP immunoreactivity was detected in the corpus callosum and periventricular white matter of pups suffering TSHI or the dual TSHI + LPS injury, compared to sham pups (Figure 3D). MBP immunolabeling of LPS brains at P15 did not differ from shams (data not shown). To further quantify the MBP loss, western blots were performed on white matter microdissected from P15 pups. Loss of MBP following *in utero* TSHI + LPS caused a 31% reduction of MBP (0.69 ± 0.04, n = 12) compared to sham levels (1.00 ± 0.08, n = 7, *P* = 0.047), whereas TSHI alone led to only a partial (13%) decrease in MBP expression (0.87 ± 0.1, n = 10, *P* > 0.05). Similar to the immunolabeling, LPS alone did not affect MBP protein expression at P15 (data not shown). At P15 no differences in NF levels, phosphoneurofilament (pNF) levels or pNF/NF ratios were found amongst the injury types on western blots (data not shown). Together, these results suggest that the combined TSHI + LPS *in utero* injury on E18 causes loss of MBP, a marker of myelination at P15, approximately equivalent to a 2-year old toddler.

### Prenatal transient systemic hypoxia-ischemia causes chronic white matter and axonal injury

**Figure 4:**
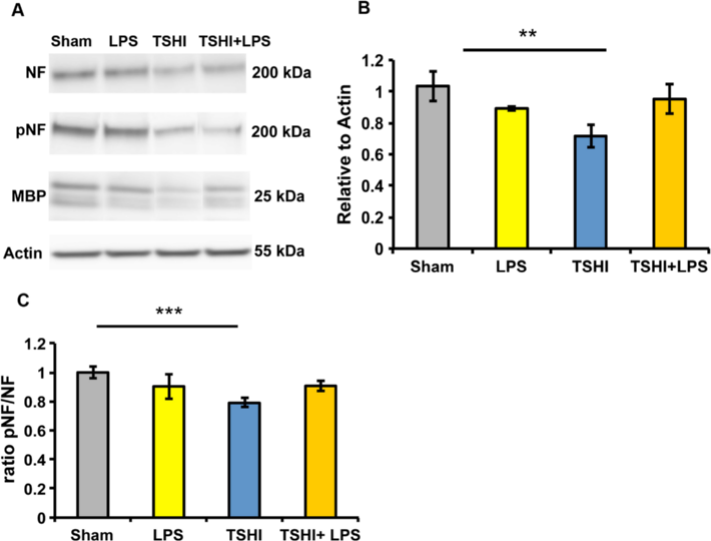
**Myelin basic protein and neurofilament expression is significantly decreased in transient systemic hypoxia-ischemia pups on postnatal day 28. (A)** Following transient systemic hypoxia-ischemia (TSHI) on embryonic day 18, western blot analyses at postnatal day 28 reveal protein expression of myelin basic protein (MBP), phosphoneurofilament (pNF), and neurofilament (NF) is significantly altered in microdissected white matter. **(B)** MBP expression is significantly decreased in microdissected white matter from TSHI pups compared to sham levels (n = 3-10, **P < 0.01). **(C)** Similarly, the ratio of pNF/NF is significantly decreased in TSHI pups compared to shams, indicative of axonal dysfunction (n = 4-13, ***P < 0.001). LPS, lipopolysaccharide.

To determine whether the reduced expression of MBP observed at P15 was transient or sustained, white matter was also analysed at P28, a time point equivalent to an older teenager when myelination is complete. These studies revealed an apparent shift from an acute white matter injury pattern with MBP loss in combined TSHI + LPS at P15 to a different chronic injury pattern at P28 juvenile rats. Notably, the chronic white matter injury pattern was present in TSHI pups, but not observed in rats exposed to LPS. At P28, TSHI pups had significantly decreased MBP protein expression compared to shams (Figure 4A). Specifically, P28 TSHI pups had a 28% reduction in MBP (0.72 ± 0.07, n = 10) compared to sham animals (1.04 ± 0.10, n = 7, *P* = 0.038, Figure 4B). Analysis of the axonal proteins, NF and pNF, corroborated MBP levels and revealed chronic axonal injury was also present in TSHI pups. The ratio of pNF/NF, a measure of axonal health, was markedly reduced at P28 in TSHI pups (0.79 ± 0.03, n = 9) compared to shams (1.00 ± 0.04, n = 6, *P* = 0.005, Figure 4C), consistent with the chronic loss of MBP observed at P28. Interestingly, these results indicate prenatal TSHI causes a more sustained white matter injury than prenatal LPS-induced inflammation, suggesting that LPS and TSHI have differing mechanisms of injury that cause varying patterns of injury to the immature white matter and developing axons during the first month of postnatal CNS maturation.

### Injury impacts erythropoietin ligand and receptor mRNA levels

**Figure 5:**
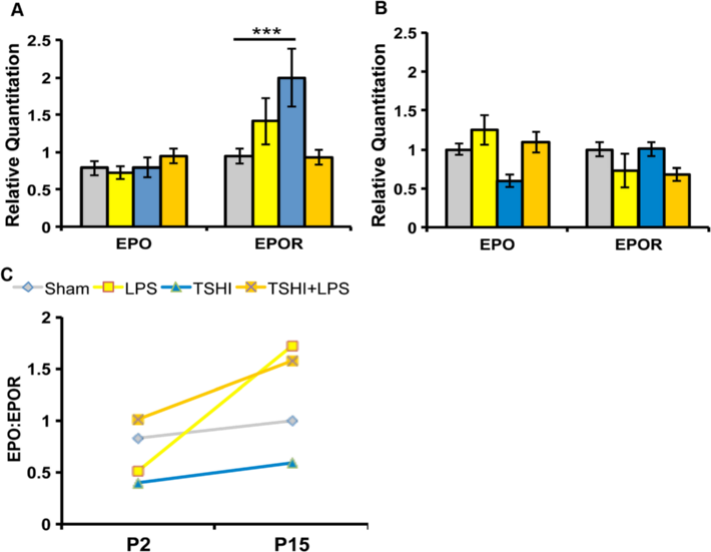
**Injury impacts erythropoietin ligand and receptor mRNA levels. (A)** At postnatal day 2 (P2), erythropoietin receptor (EPOR) mRNA levels are significantly higher in transient systemic hypoxia-ischemia (TSHI) pups compared to sham levels, without a concomitant increase in erythropoietin (EPO) ligand mRNA levels (n = 5-14, ***P < 0.001). **(B)** By P15 no significant differences in EPO or EPOR mRNA exists between injury groups (n = 3-7). **(C)** The patterns of the ratio of EPO ligand to EPO receptor shift from P2 to P15, with sustained ligand to receptor mismatch in TSHI pups, in contrast to the shift observed in pups exposed to lipopolysaccharide (LPS) or TSHI + LPS. The persistent ligand deficiency correlates with the sustained loss of myelin basic protein and the reduced phosphoneurofilament/neurofilament ratio observed at P28 in TSHI white matter, but not LPS or TSHI + LPS white matter.

EPO signaling influences the genesis, survival and maturation of developing neural cells after prenatal injury, primarily through the balance of the ligand and receptor levels, which are transcriptionally regulated [37,38]. To determine if the different *in utero* lesions altered postnatal EPO signaling in the brain, quantitative PCR analyses of EPO and EPO receptor (EPOR) mRNA were performed at P2 and P15. At P2, EPO ligand mRNA was consistent across the injured groups compared to shams (Figure 5A). In contrast, significant increases in EPOR mRNA were observed in TSHI pups (2.0 ± 0.1, n = 7) compared to sham controls (0.9 ± 0.1, n = 7, *P* = 0.001, Figure 5A). The relative levels of mRNA expression had shifted by P15 (Figure 5B). Levels of white matter EPO lig and mRNA trended down following TSHI, while in the presence of LPS-induced inflammation levels were stable, without or with TSHI. Similarly, EPOR mRNA levels were lower at P15 in the presence of LPS. As shown in Figure 5C, the pattern of the EPO ligand:receptor ratio shifted from P2 to P15. The ligand:receptor mismatch and relative ligand deficiency persisted in TSHI pups from P2 through P15, consistent with the reduced MBP expression and pNF/NF ratio observed in white matter from TSHI rats at P28. By contrast, in the presence of LPS, the EPO ligand:receptor ratio stabilized by P15, consistent with the lack of significant white matter MBP and NF changes observed in LPS or TSHI + LPS rats at P28. These data indicate that the impact of prenatal injury on EPO signaling may vary depending on whether concurrent inflammation is present or not, and that prenatal TSHI may induce a more severe, sustained injury to the developing white matter. Further detailed analyses are underway to clarify these complex interactions.

### Prenatal injury impacts motor function in juvenile rats

**Figure 6:**
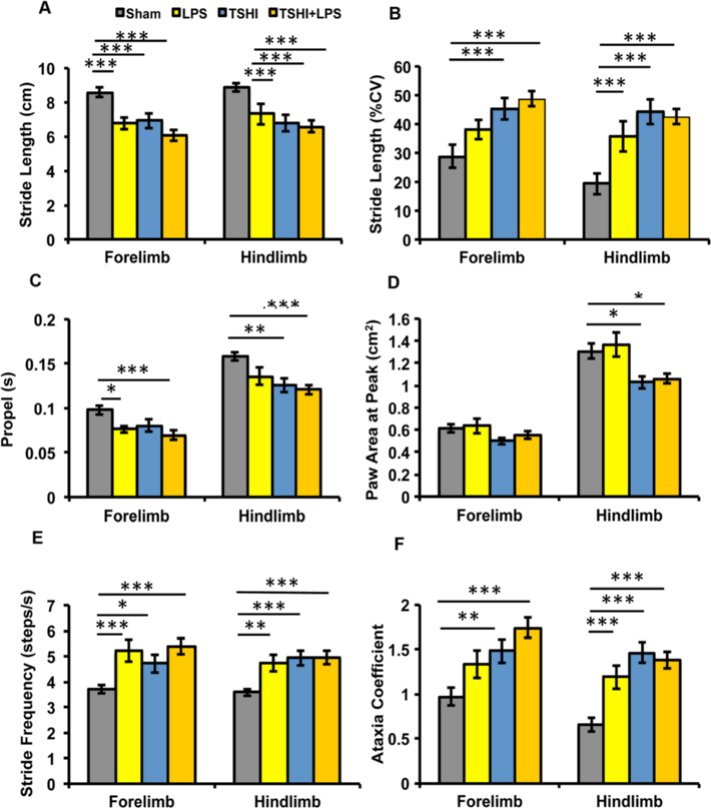
**Prenatal injury significantly impacts motor function in juvenile rats.** Digigait analyses at postnatal day 28 demonstrate significant motor impairment and gait abnormalities in both forelimbs and hindlimbs of lipopolysaccharide (LPS; n = 13), transient systemic hypoxia-ischemia (TSHI; n = 14) and TSHI + LPS (n = 21) rats, compared to shams (n = 18). Impairment includes **(A)** decreased stride length, **(B)** increased stride variation, **(C)** decreased time in the propel phase, **(D)** decreased paw area at peak stride, consistent with toe-walking, and **(E)** increased stride frequency, which all culminate in **(F)** increased ataxia coefficients. *P < 0.05, ** *P* < 0.01, *** *P* < 0.001, versus shams. CV, Coefficient of variation.

Consistent with the clinical picture of cerebral palsy and spasticity in children suffering perinatal brain injury, LPS (n = 13), TSHI (n = 14) and TSHI + LPS (n = 21) rats showed functional gait abnormalities and postural instability, including significant impairments in stride, cadence, consistency and coordination compared to shams (n = 18; Figure 6). Cumulatively, movement of the forelimbs and hindlimbs were abnormal, and most rats with prenatal injury with all lesion types had difficulty with coordinated stepping, as indicated by the reduced stride length (Figure 6A), an increased coefficient in stride length variation (Figure 6B), stride frequency (Figure 6E) and ataxia (Figure 6F). Also, hindlimb time in the propel phase was reduced in TSHI and TSHI + LPS pups (Figure 6C). Interestingly, TSHI and TSHI + LPS pups exhibited evidence of toe walking consistent with spastic gait hallmarked by significantly decreased paw area at peak stance (Figure 6D). Similar to children with spasticity from prematurity, the hindlimbs were affected more severely than the forelimbs for most parameters, including the paw area (Figure 6D). While all injuries markedly affected the gait, the presence of TSHI, with or without LPS, produced more abnormalities. Subgroups analyses for sex did not reveal sex-specific differences in the deficits, except that females did not have decreased time in the propel phase, and males did not have decreased paw area at peak stance for any of the injury groups. Together, these gait studies in juvenile rats demonstrate that prenatal injury induces persistent gait abnormalities with similarities to the gait abnormalities found in children with spasticity from preterm birth. More importantly, these functional studies suggest that the various types of prenatal injuries result in different gait abnormalities.

## Discussion

Exposure of preterm infants to perinatal infection and inflammation is associated with an increased risk for neonatal brain damage and developmental disabilities [6,7,9]. Here, using a clinically relevant rat model mimicking brain damage from extreme preterm birth, we demonstrate for the first time that the combination of *in utero* TSHI and intra-amniotic LPS-induced inflammation results in cellular, biochemical and functional abnormalities in 1 month old rats. Moreover, we found that the various types of prenatal injuries have differing, but consistent and coherent patterns of impact on white matter development, gait abnormalities and EPO signaling.

While it has been recognized for many years that inflammatory stimuli contribute to perinatal brain injury, the pathophysiological mechanisms and models designed to uncover those mechanisms have varied. Recent reports indicate that chorioamnionitis can lead to a fetal inflammatory response syndrome promoting a global inflammatory reaction and is a significant risk factor for multiple complications in the preterm infant [44,45]. In order to recapitulate the common clinical occurrence of placental inflammation from intrauterine bacteria and HI associated with very preterm birth, we added intra-amniotic LPS administration to our previously published rat model of prenatal TSHI that mimics injury at approximately 25 weeks EGA [33]. LPS is a non-infectious cell wall component of Gram-negative bacteria that has previously been introduced through various routes to induce inflammation in the developing brain [24-26,46]. Activation of toll-like receptor 4 is triggered by LPS in a developmentally regulated manner [47], and is essential for initiation of neuronal injury from intrauterine LPS exposure [48]. We found that the addition of intra-amniotic LPS to *in utero* transient global HI on E18 results in approximately 44% fetal loss and causes encephalomalacia with ventriculomegaly, and reactive astrocytosis and microgliosis at P2, 1 week after the prenatal injury and equivalent to approximately 30 to 32 weeks EGA. These acute macroscopic and microscopic findings are consistent with those observed in preterm infants with white matter lesions [35,49,50].

By isolating the different types of injuries, we found the pattern of white matter damage is complex and evolves during the first postnatal month. While the ventriculomegaly and acute gliosis diminishes in this model by P15, the approximate midpoint of myelination, loss of MBP protein becomes apparent at P15 following both TSHI alone and TSHI + LPS. At P15 the MBP loss is more pronounced following the combined prenatal TSHI + LPS injury than with TSHI alone. Interestingly, analysis of juvenile rats at P28 indicated a shift in white matter injury patterns whereby MBP loss persisted and worsened in animals subjected to TSHI alone, consistent with prior studies that showed prenatal TSHI causes sustained loss of myelin in adults rats [33,37,51], and hinders multiple stages of oligodendroglial lineage development [33,38]. The MBP loss following prenatal TSHI was accompanied by reduced pNF/NF ratio, consistent with axonopathy observed in humans [52,53] and rats with white matter lesions from prenatal ischemia [54]. By contrast, MBP loss stabilized at P28 in animals with LPS present, and no reduction of pNF/NF occurred. Together, these results support the hypothesis that prenatal LPS-induced responses alter the pattern of white matter injury differently compared to TSHI. These evolving patterns of white matter injury during development are also consistent with prior studies that showed two phases of inflammatory response induced by LPS [55]. The evolving pattern of white matter injury in this rodent model replicates the dysmaturation of white matter observed in recent human imaging studies [56,57].

We next investigated whether the different patterns of white matter injury induced by various prenatal injuries correlated with functional outcomes using a detailed analysis of gait, since motor impairment manifesting as cerebral palsy, or developmental coordination disorder is a common deficit in children born preterm [58]. In humans, gait dynamics extend beyond measure of stride length and, thus, we investigated additional metrics including spatial and temporal gait indices [42]. Here, we report chronic motor impairment, decreased stride length, increased stride variation and decreased paw area in hindlimbs consistent with toe-walking. As step-to-step gait variability is common in humans with cerebral palsy, spasticity and abnormalities of tone, we investigated ataxia coefficients and movement through the midline (an indication of wobble and unsteadiness). We found rats subjected to intra-amniotic LPS alone, TSHI alone and TSHI + LPS each had significantly increased ataxia coefficients, increased stance variability and decreased stride lengths. This postural instability and reductions in stance duration may result in a shorter time for limb muscles to be activated for stabilization [42,59], and may account for the significant increases in stride-to-stride variability observed in the injured animals. Consistent with previous reports [43], and the clinical picture of spasticity and cerebral palsy in children who are born preterm, measures of hindlimb incoordination display more ataxic perturbation than forelimbs. Stride length may be determined predominantly by a gait-patterning mechanism, whereas stance width may be determined by a balance-control mechanism [42,60]. Although all three prenatal injuries (LPS alone, TSHI alone and TSHI + LPS) caused similar gait deficits in stride, posture and balance, the presence of TSHI added additional deficits indicative of toe-walking, reminiscent of the spastic gait observed in children born preterm. The additional deficits found with TSHI are consistent with the evidence of impaired myelination and axonopathy discovered in our biochemical analyses at P28. While caution must be used in extrapolating from biochemical analyses and gait studies in young rats, our results support the hypothesis that the mechanisms of white matter injury induced by HI and bacterial inflammation likely overlap only partially.

EPO has multiple mechanisms of neuroprotection that may be beneficial to preterm infants, in part due to the importance of EPO signaling during CNS development and recovery from injury [61-63]. Indeed, a recent multi-center trial demonstrated that very preterm infants born at <1,250 g who were treated with erythropoiesis-stimulating agents showed better cognition at 2 years compared to placebo-treated infants [64]. Injury can induce an excess of EPOR expression that is not matched by a concomitant increase in ligand, resulting in excess neural cell apoptosis [37]. Exogenous EPO treatment restores the relative deficit in EPO ligand, mitigates the EPO ligand:receptor mismatch and redirects neural cells to survival and maturation [37,38]. Most studies in rodents demonstrating sustained efficacy of EPO treatment in the mature CNS following perinatal injury have used HI [37,65-68]. EPO treatment has also shown long-term efficacy in improving cognitive outcomes in rats with bacterial meningitis [69], but no studies have investigated the efficacy of EPO treatment in a dual injury model. Consistent with the different patterns of white matter injury and impaired gait with the different types of lesions observed here, the ratio of EPO ligand to receptor was impacted differently by the various prenatal insults over the first 3 postnatal weeks. Following prenatal TSHI the ratio of EPO:EPOR remained below sham levels from P2 through P15, consistent with the significant ligand deficit relative to receptor that has previously been found with prenatal TSHI [37]. By contrast, the ratio of EPO:EPOR improved between P2 and P15 for the rats exposed to prenatal LPS. This pattern change may correlate with the less severe white matter injury observed at P28 in rats exposed to LPS or TSHI + LPS, compared to TSHI alone, which showed sustained loss of MBP expression and reduced pNF/NF ratios. Consistent with the milder white matter injury observed with the biochemical analyses at P28 in the rats exposed to LPS, the gait abnormalities in the LPS-exposed rats were also slightly milder. These milder biochemical and functional deficits coincided with slightly higher EPO ligand and lower EPOR transcription at P15 in the presence of LPS. Prior studies have found differences in the inflammatory response depending on the age of the animal at the timing of the LPS and HI exposure [24,27,47,55], which may be related to the maturation of the toll-like receptor 4 signaling [47]. Here a combined intrauterine insult was used to mimic a dual prenatal insult that affects ELGANs, and compared to isolated prenatal LPS and HI insults. More extensive analyses are needed to clarify whether the secondary phase of the response to prenatal LPS exposure is less harmful than prenatal HI. For numerous reasons care must be taken with extrapolation from rodents to human neurodevelopment, especially because of the critical role of the subplate in human cortical development, and the dramatic differences in the subplate between humans and rodents [70,71]. These data suggest, however, that the impact of prenatal injury varies depending on whether inflammation is present or not, and that early HI may induce a more severe, persistent injury to the developing white matter. Together, these results support the development of specific clinically feasible and safe biomarkers to guide the use of emerging neuroprotective agents for critically ill preterm infants in real-time. Preterm infants who suffer HI may need a different treatment paradigm than those exposed to bacterial inflammation, or a combined injury. Indeed, hypothermia, a neuroprotective therapy used in term infants with HI injury, was found not to be effective when P7 rat pups were pretreated with LPS prior to the HI insult [72]. Additional studies are also needed to clarify the impact of cumulative postnatal HI or infectious insults on the developing brain. Clarification of injury mechanisms with clinically relevant models such as presented here will advance our treatment regimens and ultimately improve outcomes for ELGANs.

## Conclusions

Prenatal LPS, TSHI and TSHI + LPS lead to different patterns of injury with respect to white matter injury, as shown by myelination and axon integrity, gait deficits and EPO signaling. Dual injury leads to acute alterations in glial response and cellular inflammation, while TSHI alone causes chronic white matter and axonal injury. Both injuries cause significant gait deficits, although with different patterns. Both injuries also alter the transcriptional regulation of EPO ligand and receptor during the neonatal period. Further study will contribute to stratification of injury mechanisms in preterm infants, and guide the use of promising therapeutic interventions.

## Abbreviations

ANOVA: analysis of variance; CNS: central nervous system; E(x): embryonic day (x); EGA: estimated gestational age; ELGAN: extremely low gestational age newborn; EPO: erythropoietin; EPOR: erythropoietin receptor; GFAP: glial fibrillary acidic protein; H&E: hematoxylin and eosin; HI: hypoxia-ischemia; Iba1: ionized calcium-binding adapter molecule 1; LPS: lipopolysaccharide; MBP: myelin basic protein; NF: neurofilament; P(x): postnatal day (x); PCR: polymerase chain reaction; pNF: phosphoneurofilament; ROI: regions of interest; TSHI: transient systemic hypoxia-ischemia.

## Competing interests

The authors declare that they have no competing interests.

## Authors’ contributions

LLJ contributed to hypothesis development, interpreted data, wrote the manuscript and performed the quantitative PCR, western blot, biochemical and digigait studies. CJC performed the surgeries, completed P15 stereology studies and contributed to the gait analyses and data acquisition. JB performed P2 stereology studies and contributed to the gait analyses and data acquisition. DJF performed surgeries and conducted P2 stereology studies. JF participated in P2 stereology data collection. RM contributed to hypothesis development, data interpretation and manuscript preparation. SR conceived the hypothesis, supervised all portions of data collection, analysis and interpretation, and manuscript preparation. All authors read and approved the final manuscript.
